# Peripheral neuron phenotypes of familial dysautonomia are rescued by AAV-mediated gene therapy

**DOI:** 10.21203/rs.3.rs-7529031/v1

**Published:** 2025-09-23

**Authors:** Nadja Zeltner, Hsueh-Fu Wu, Tripti Saini, Jennifer Art, William Delaney, Frances Lefcort

**Affiliations:** University of Georgia; University of Georgia; University of Georgia; University of Georgia; University of Georgia; Montana State University

## Abstract

Familial dysautonomia (FD) is a rare genetic, neurodevelopmental and neurodegenerative disorder, where a homozygous mutation in the *ELP1* gene is responsible for defects and symptoms found in 99% of patients (1). FD symptoms mainly affect the peripheral nervous system (PNS) (2), including the autonomic and sensory nervous systems (ANS, SNS) (3). The ANS regulates unconscious physiological responses and maintains body homeostasis, such as heart rate, blood pressure, gland secretion, and breathing, which are vital for bodily function. The SNS is the key mediator that processes and relays sensory information from the internal and external environment to the brain, including limb position, temperature, and pain.

Familial dysautonomia (FD) is a rare genetic, neurodevelopmental and neurodegenerative disorder, where a homozygous mutation in the *ELP1* gene is responsible for defects and symptoms found in 99% of patients (1). FD symptoms mainly affect the peripheral nervous system (PNS) (2), including the autonomic and sensory nervous systems (ANS, SNS) (3). The ANS regulates unconscious physiological responses and maintains body homeostasis, such as heart rate, blood pressure, gland secretion, and breathing, which are vital for bodily function. The SNS is the key mediator that processes and relays sensory information from the internal and external environment to the brain, including limb position, temperature, and pain.

Both ANS and SNS are defective in FD (1). Patients suffer from dysautonomic symptoms, such as dysregulation of heart rate and blood pressure, flushing, and abnormal sweating. Physical or emotional stress can further trigger life-threatening episodes called dysautonomic crises, characterized by hypertension, vomiting/retching, and personality changes (1). Postganglionic sympathetic neurons (symNs) of the ANS, which control the fight-or-flight response and increase autonomic functions, have been found to be decreased by about 30% in FD patients (2). This is accompanied by sympathetic hyperexcitability and norepinephrine (the main neurotransmitter of symNs) overspill (1). FD patients also have difficulties sensing temperature/pain and suffer from gait ataxia, which is attributed to reduced sensory neuron numbers in patients’ sensory ganglia (2). While FD patients are born with symptoms, for example, difficulties swallowing, which leads to problems with feeding, research over the past decades has shown that PNS neurons also degenerate over time, leading to worsening of symptoms throughout life (1). FD is also marked by the progressive death of retinal ganglion cells (4).

Human pluripotent stem cells (hPSCs), including embryonic stem cells (ESCs, derived from the blastocyst) and induced pluripotent stem cells (iPSCs, reprogrammed from adult somatic cells), have been prosperously applied to study human diseases in recent years (5). hPSCs can self-renew *in vitro*, which provides an unlimited source of human, untransformed cells for research. By differentiating hPSCs, derived from a patient, into the desired cell types that are affected in the patient, scientists are able to model specific cellular and molecular defects contributing to the disease (5). This technology, however, rests on the premise that reliable differentiation protocols to produce the desired cell types have been established. We have developed highly efficient, stable, and reproducible methods to differentiate multiple PNS lineages from hPSCs, including symNs, parasympathetic neurons and sensory neurons (6–8). We employed these tools to study FD disease mechanisms using healthy and FD patient-derived hPSC models. We found that in FD, the PNS progenitor neural crest cell development was significantly impaired, leading to reduced neuron numbers in the sympathetic and sensory lineage (6, 9). We further described that FD symNs displayed spontaneous hyperactivity (6), which was rescued by some currently used FD treatments and novel compounds. Using this model, we further described severe developmental and neurodegenerative defects in FD sensory neurons, which allowed us to conduct drug screening and drug discovery (9). Our hPSC-based FD models, therefore, have been shown to be a critical tool to assess potential therapies for FD.

ELP1 belongs to the transcriptional elongator complex, which regulates tRNA modification; thus, it is critical for both transcription and translation (10). The founder point mutation in the splice site of intron 20 of *ELP1* in FD results in a significant decrease of functional ELP1 RNA and protein (1). Recent gene therapy approaches utilizing antisense oligonucleotides (ASOs) and small nuclear RNAs have shown promising results in correcting *ELP1* expression, which may rescue or mitigate FD disease phenotypes (11, 12). Broadly, ASOs are short, single-stranded DNA/RNA analogs with the ability to bind to target RNA and modulate splicing signals on pre-mRNA (13). In FD, they work by targeting the mutation on *ELP1* mRNA and correcting the defective splicing, potentially restoring cellular abnormalities (14). Recently, Schultz et al. used adeno-associated virus (AAV) in the FD mouse model, where progressive death of retinal ganglion cells and their axon degeneration occurs. Similar to the FD patient phenotype, which leads to vision loss later in life. They sought to use gene replacement therapy of *ELP1* to treat the optic neuropathy in FD mice. AAVs were used to deliver a functional murine *Elp1* gene (AAV2-U1a-*Elp1*) by intravitreal injections (15). As a result, they observed significantly improved retinal ganglion cell survival in FD mouse eyes compared to the untreated group. The eGFP control group (AAV2-U1a-eGFP) also showed significant protective effects albeit less than the murine Elp1-AAV2 group (15).

These exciting breakthroughs lead us to test the therapeutic effects of this gene replacement approach in our human FD PNS models. We first asked whether gene therapy can rescue FD symN hyperactivity (6). AAV carrying healthy human *ELP1* (AAV2-U1a-h*ELP1*) was added to FD hPSC-derived symNs on day 20 of differentiation (Fig. 1A, top), the stage when symN differentiation is complete, while the FD hyperactivity phenotype is not yet observed (it is detected after day 30) (6). Experiments were performed on 96-well multielectrode array (MEA, by AxionBiosystems) plates with transparent bottoms (Fig. 1B), enabling us to monitor both the efficiency of viral transduction and neural activity at the same time. For each experimental group and each biological repeat (defined as independent differentiations), 6×96-well MEA wells were used and averaged to avert well-to-well variability. On day 20 of differentiation, we treated FD symNs with AAV2-U1a-h*ELP1* or AAV2-U1a-*eGFP* control, respectively, at an MOI of 400,000 (2.7×10^12^ vg/ml for AAV2-U1a-*ELP1* and 8.8×10^12^ vg/ml for AAV2-U1a-*eGFP*), in 30 μl symN culture medium/96 well. 6 hours after the transduction, 120 μl fresh symN culture medium was added to each well. The next day (day 21), a complete medium change was performed to remove the AAV (Fig. 1A). On day 30, we compared the neural activity of untreated AAV2-U1a-*ELP1* or AAV2-U1a-*eGFP* treated FD symNs to healthy symNs and performed additional assays. On day 30, AAV2-U1a-*eGFP* treated FD symNs, were successfully transduced, where about 60% of total cells were eGFP+ (Fig. 1B and 1C). All symN groups showed a similar level of cell density (Fig. 1D), which excludes the possibility that the difference in neural activity is caused by uneven cell numbers or possible viral toxicity. We next examined the level of *ELP1* mRNA splicing via RT-qPCR in each group. Compared to untreated and AAV2-U1a-*eGFP*-treated FD symNs, AAV2-U1a-*ELP1*-treated FD symNs showed a dramatic rescue effect in *ELP1* splicing, not significantly different from healthy symNs (Fig. 1E). Lastly, we analyzed neural activity among each group by MEA. Compared to healthy symNs, untreated FD symNs were hyperactive as previously reported (6) AV2-U1a-*ELP1* treatment significantly reduced this hyperactivity phenotype, to levels of the healthy control (Fig. 1F). Interestingly, similar to the results by Schultz et al., we also observed a mild level of rescue effect in AAV2-U1a-*eGFP* treated FD symNs, although not significantly different from untreated FD (Fig. 1F). The similarity of the rescue effect on FD iPSC-derived symNs and FD mouse retina again highlights the utility and reliability of hPSC-based disease modeling on identifying tissue specific pathologies and novel treatments in neurological disorders. Taken together, our data suggest that AAV2 gene therapy rescues the hyperactivity phenotype in human FD symNs.

We next aimed to assess if these positive effects of AAV2-U1a-*ELP1* on FD are also present during sensory neuron (SN) development. SN differentiation was performed on regular 96-well plates. On day 14 of differentiation, at the early immature SN stage, the FD groups were transduced with AAV2-U1a-*ELP1* or AAV2-U1a-*eGFP* at the same MOI of 400,000 (2.7×10^12^ vg/ml for AAV2-U1a-*ELP1* and 8.8×10^12^ vg/ml for AAV2-U1a-eGFP). On day 30, we observed that the SNs were successfully transduced at this concentration, but the cells were stressed in both AAV2-U1a-*ELP1* or AAV2-U1a-eGFP control treatments (Fig. 1G, top). By performing RT-qPCR, we also observed that *ELP1* expression was very high in AAV2-U1a-*ELP1* (Fig. 1H). Thus, we next treated the FD neurons using an MOI of 200,000 (1.35 × 10^12^ vg/ml for AAV2-U1a-*ELP1* and 4.4 ×10^12^ vg/ml for AAV2-U1a-eGFP). On day 30, we confirmed effective viral transduction by observing eGFP expression in AAV2-U1a-*eGFP*-treated FD SNs and the cells were not stressed (Fig. 1G, bottom). RT-qPCR analysis confirmed *ELP1* splicing rescue in AAV2-U1a-*ELP1* transduced FD SNs (Fig. 1I). Our data suggest that AAV2 gene therapy rescues FD *ELP1* deficiency in human FD SNs.

Together, our data show that hPSC-based disease modeling can be used as a reliable human platform to assess potential rescue effects of AAV-based gene therapy approaches. We demonstrate the promising therapeutic effect of AAV2-based gene therapy in both SNs and symNs, supporting it as a treatment for FD.

## Figures and Tables

**Figure 1:**
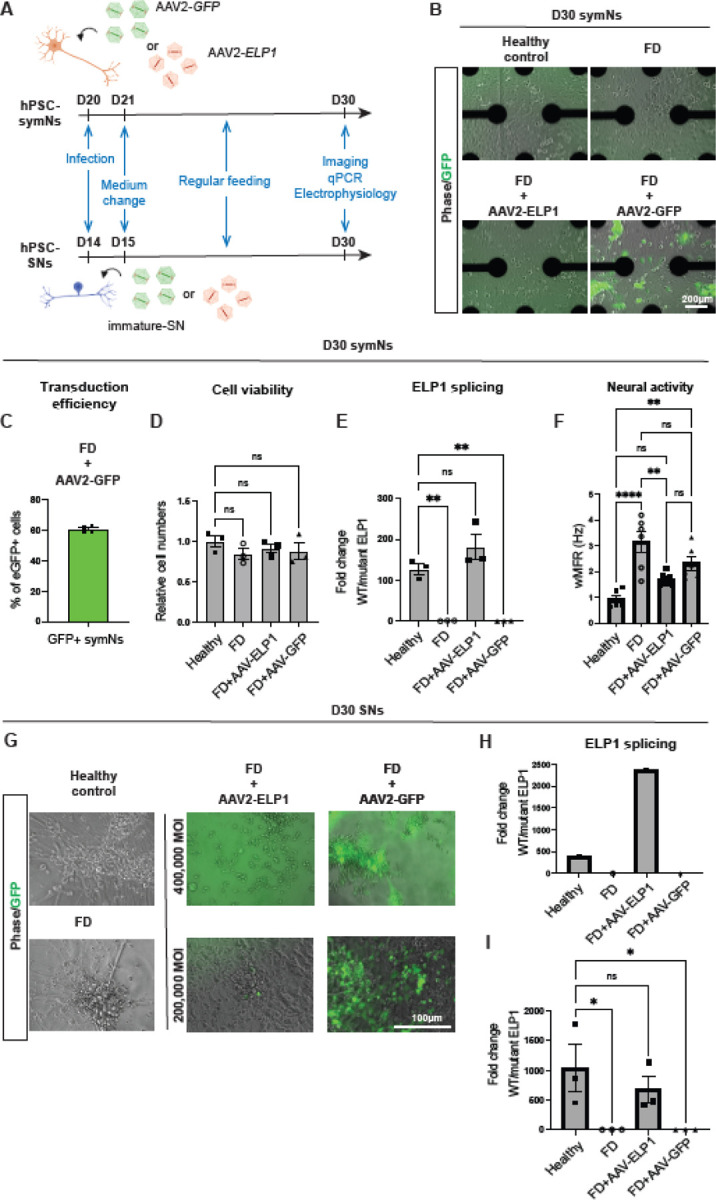
Examination of AAV-based gene therapy in human PNS neurons in FD. **(A)** Schematic cartoon showing the timeline of AAV treatments in FD sympathetic (symNs) and sensory neurons (SNs). NC=Neural crest. **(B)** Phase contrast and fluorescent images show symN morphology and eGFP expression/transduction of the neurons plated on MEA plates and assessed on day 30. Black strings/dots in the wells are the electrodes of the MEA plate. **(C)** The Y axis shows the percentage of eGFP+ cells of the total number of cells. **(D)** SymN cell numbers per well, relative to the non-transduced, healthy symNs. One-way ANOVA followed by Tukey’s multiple comparisons, where ns is non-significant, * is p£ 0.05, ** is p£ 0.01, *** is p£ 0.001, **** is p£ 0.0001. **(E)** RT-qPCR analysis for the ratio of wild type to mutant *ELP1* splicing in symNs. One-way ANOVA followed by Tukey’s multiple comparisons, n=3 biological replicates, where ns is non-significant, * is p£ 0.05, ** is p£ 0.01, *** is p£ 0.001, **** is p£ 0.0001. **(F)** MEA-based electrophysiological activity comparison. Data is presented as the weighted mean firing rate (wMFR, Mean firing rate multiplied by the number of active electrodes in a well). One-way ANOVA followed by Tukey’s multiple comparisons, n=6 biological replicates, where ns is non-significant, * is p£0.05, ** is p£ 0.01, *** is p£ 0.001, **** is p£ 0.0001. **(G)** Phase contrast and fluorescent images showing the SN morphology and eGFP expression at 400,000 and 200,000 MOI’s on a regular 96-well plate, on day 30 of SN differentiation. **(H)** RT-qPCR analysis for the ratio of wild type to mutant *ELP1* splicing in SNs at MOI 400,000. n=1 biological replicate. **(I)** RT-qPCR analysis for the ratio of wild-type to mutant *ELP1* splicing in SNs at 200,000 MOIs. One-way ANOVA followed by Tukey’s multiple comparisons, n=3 biological replicates, where ns is non-significant, * is p£0.05, ** is p£ 0.01, *** is p£ 0.001, **** is p£ 0.0001.
